# Quantitative In Vivo Magnetic Resonance Spectroscopy Using Synthetic Signal Injection

**DOI:** 10.1371/journal.pone.0015166

**Published:** 2010-12-28

**Authors:** Kenneth I. Marro, Donghoon Lee, Eric G. Shankland, C. Mark Mathis, Cecil E. Hayes, Seth D. Friedman, Martin J. Kushmerick

**Affiliations:** 1 Department of Radiology, University of Washington, Seattle, Washington, United States of America; 2 Department of Physiology and Biophysics, University of Washington, Seattle, Washington, United States of America; 3 Department of Radiology, Seattle Children's Hospital, Seattle, Washington, United States of America; Cuban Neuroscience Center, Cuba

## Abstract

Accurate conversion of magnetic resonance spectra to quantitative units of concentration generally requires compensation for differences in coil loading conditions, the gains of the various receiver amplifiers, and rescaling that occurs during post-processing manipulations. This can be efficiently achieved by injecting a precalibrated, artificial reference signal, or pseudo-signal into the data. We have previously demonstrated, using in vitro measurements, that robust pseudo-signal injection can be accomplished using a second coil, called the injector coil, properly designed and oriented so that it couples inductively with the receive coil used to acquire the data. In this work, we acquired nonlocalized phosphorous magnetic resonance spectroscopy measurements from resting human tibialis anterior muscles and used pseudo-signal injection to calculate the Pi, PCr, and ATP concentrations. We compared these results to parallel estimates of concentrations obtained using the more established phantom replacement method. Our results demonstrate that pseudo-signal injection using inductive coupling provides a robust calibration factor that is immune to coil loading conditions and suitable for use in human measurements. Having benefits in terms of ease of use and quantitative accuracy, this method is feasible for clinical use. The protocol we describe could be readily translated for use in patients with mitochondrial disease, where sensitive assessment of metabolite content could improve diagnosis and treatment.

## Introduction

One of the most enticing features of magnetic resonance spectroscopy (MRS) is its feasibility for use as a noninvasive biochemical assay. This potential arises from the fact that the amplitudes of the peaks in the processed spectra are directly proportional to the number of excited nuclei within the measurement volume. Unfortunately, the proportionality is also affected by a variety of other parameters such as pulse sequence timing parameters and their interaction with relevant relaxation times, B_1_ field homogeneity and its effects on signal excitation and reception sensitivity, etc. The level of diligence required to measure and/or compensate for all of these parameters is technically challenging for research sites [1], [2] and beyond the capabilities of most clinical sites so nearly all MRS measurements are reported as relative changes or in terms of ratios, which can be difficult to interpret.

When making the conversion to units of concentration, one of the most cumbersome parameters to address is RF coil loading, which determines the amplitude of the current generated in the coil used to receive the MR signal (and therefore the subsequent amplitude of the peak in the spectrum) per unit of excited nuclei in the sample volume. Coil loading is dependent on the electrical properties of the coil, the geometry of the magnetic flux lines the coil produces, and the concentration and distribution of electrolytes in the sample. It is difficult to measure accurately and can vary substantially between subjects or even for the same subject depending on the relative position of the coil.

In 1997, Barantin, et al [3] demonstrated how an artificially injected reference signal can be used to compensate for coil loading conditions. The method they developed, Electronic REference To access In vivo Concentrations (ERETIC), uses either a broad band antenna [3], [4] or the second coil in a high resolution probe [5]–[7] to broadcast an amplitude modulated signal that replicates a free induction decay (FID). Radiation coupling between the antenna and the RF coil creates an artificial peak, or pseudo-peak, in the processed spectrum. The amplitude of the pseudo-peak is first calibrated using a phantom with a known concentration of an MR-visible molecule. Transmission of the same pseudo-signal during subsequent measurements creates a scale factor that is used to convert peak amplitudes to units of concentration. This method can be applied to any MR-visible nucleus. The user sets the amplitude, frequency, phase, and line-width of the pseudo-peak as desired. The frequency is then set to a convenient location, within the acquisition bandwidth, but not overlapping real peaks. The electronically injected reference signal is readily available and eliminates the need to maintain stable biochemical samples for use as external references.

Implementation of the ERETIC method in humans is hindered by the use of radiation coupling to inject the artificial signal. A broadcasted signal can reflect off, or be absorbed by, objects in the magnet room. A change in position of a reflector or absorber, including the sample itself, could alter the coupling between the transmitter and the receive coil and change the factor of proportionality established during the calibration session. This places constraints on implementation that are impractical for clinical sites so, while the original ERETIC measurements were reported in humans [3] nearly all subsequent implementations were in vitro [4]–[25] or in small animals [26], where the sample size was small and consistent. The recent work of Heinzer-Schweizer, et al. [27] is a notable exception to this pattern.

We have developed a method that provides the same advantages as the ERETIC method but creates a more stable reference signal, making it suitable for human studies. Instead of radiation coupling, we use inductive coupling to inject the pseudo-FID (pFID). This requires an additional RF coil, called the injector coil, placed close to the receive coil and fixed in position so that the overlap of the flux lines for the two coils remains constant. Current passing through the injector coil creates a magnetic field, B_1ref_, that couples inductively with the main RF coil to create the pseudo-peak. Since inductive coupling is the same mechanism by which the magnetic field arising from excited nuclei in the sample couples with the receive coil, all subsequent manipulations of the data have an equal effect on the real and pseudo-signals.

We have previously conducted in vitro validation measurements for spectroscopy [28] and imaging [29] applications. In this work we apply the pFID method to quantify inorganic phosphate (Pi), phosphocreatine (PCr) and adenosine triphosphate (ATP) concentrations using ^31^P spectroscopy measurements from resting human tibialis anterior muscles. For these initial measurements in humans, we implemented a protocol that imposed constraints on the subject pool and the acquisition parameters. These constraints were not dictated by the method we have developed but rather were imposed to allow unambiguous validation against a more established quantification method. Hence, while this work should be considered developmental, it paves the way for more sophisticated measurements in humans.

Our results reveal strong concordance between concentrations obtained using the pFID method and those obtained using the phantom replacement method [30]–[33], demonstrating feasibility of our method for human applications. We also demonstrate that the pFID method automatically compensates for differences in coil loading conditions and so allows more expedient quantification of metabolite content. We discuss how this method might be applied to patients with mitochondrial disease and other disorders.

## Methods

Prior approval for this study was obtained from the University of Washington's Human Subject Division. Written, informed consent was obtained from each subject in accordance with institutional protocol.

All experiments were conducted on a 4.7 T Bruker horizontal bore magnet equipped with a Varian Inova spectrometer and VNMR version 6.1. Before each measurement, the tune and match capacitors were adjusted to yield 50 ohms impedance and the B_o_ field was optimized by manually adjusting the shims. To ensure consistent flip angles for all measurements, the transmitter power was set to maximize the PCr signal with an RF pulse duration of 110 µsec. RF homogeneity concerns were addressed by acquiring all measurements from samples that were much larger than the sensitive volume of the surface coil and by using spacers to ensure that the separation distance between the surface coil and the measurement volume was the same for the phantom measurements as it was for the in vivo measurements.

### Probe design

**Figure 1:**
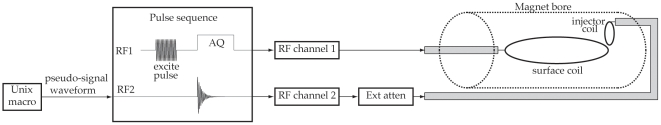
The key hardware components required to inject the pseudo-signal. Prior to execution of the pulse sequence, a Unix macro was used to create a digitized waveform describing the desired pseudo-signal. The pulse sequence read the waveform and sent it to the second RF channel (RF2). The pseudo-signal passed through an external attenuator (Ext atten) before being fed to the injector coil. During sequence execution, the surface coil was operated in transmit/receive mode while the injector coil was used only to transmit the pseudo-signal during the acquisition window (AQ).

Figure 1 contains a schematic of the hardware configuration used for all measurements. A custom-built RF probe was used to inject the pseudo-signal via inductive coupling. Details of the probe design have been described previously [28]. In brief, the probe consisted of a 20 mm diameter surface coil and a 1.5 mm diameter, 2-turn injector coil, both formed from copper wire. The main coil, which we will refer to as the surface coil, was tunable to both ^1^H and ^31^P frequencies, 200.4 and 81.2 MHz respectively, and was operated in both transmit and receive modes. The smaller coil, which we call the injector coil, was used solely to inject the pseudo-signal into the surface coil during data acquisition. The geometry and orientation of the injector were designed to minimize coupling between the injector coil and the sample. The two coils were fixed in position relative to each other, with approximately 1 mm separation distance between them, so that the overlap between their flux lines was constant for all measurements.

### Data acquisition and processing

The data were acquired using a modified version of the Varian SPULS pulse sequence and the equipment configuration (RF synthesizer, RF amplifier, cabling, etc.) typically used for spectroscopy. Acquisition parameters included repetition time (TR) = 15 sec, sweep width = 10 kHz, block size  = 4096 points. The long TR ensured that all metabolites were fully relaxed, eliminating the need to measure and compensate for longitudinal (T1) relaxation. SPULS is a nonlocalized pulse sequence with only 10 µsec delay between the excitation pulse and data acquisition, eliminating the need to measure and compensate for transverse (T2) relaxation. The pulse sequence was modified to allow transmission, via a second RF channel, of a pFID to the injector coil during the data acquisition window. The pFID was a monoexponential decay function with a time constant of 60 msec. It was digitized with 4096 complex points, the same number used for real data acquisition, and written to a text file that complied with the Varian RF waveform format. To reduce the amplitude of the B_1_ field created by the injector coil to approximately the same amplitude as the field created by excited nuclei within the acquisition volume, a 60 dB attenuator was inserted between the second RF channel and the injector coil. To eliminate radiation coupling between the injector coil circuit and the receive coil circuit, semi-rigid cabling, with two layers of solid copper shielding, was used to connect both the injector coil and surface coil.

The following equation was used to determine metabolite content [3]:
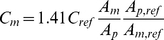
(1)where: C_m_ is the average in vivo metabolite concentration within the measurement volume, C_ref_ is the volumetrically-determined concentration of the metabolite analog used during the calibration session (see below), A_m_ and A_p_ are the amplitudes of the metabolite and pseudo-signal peaks, respectively, acquired during the in vivo session, and A_m,ref_ and A_p,ref_ are the amplitudes of the metabolite analog and pseudo-signal peaks, respectively, acquired during the calibration session. The scale factor, 1.41, was used to convert the units from mmol/L of muscle tissue to mmol/L of cellular water [34]. The amplitudes of the spectral peaks were determined using the Advanced Method for Accurate, Robust and Efficient Spectral (AMARES) time domain fitting algorithm [35] as included in the Java-based Magnetic Resonance User Interface (jMRUI) software package [36].

In vivo data were acquired from 5 normal male subjects whose subcutaneous lipid layer thickness above the tibialis anterior compartment was between 3.5 and 4.7 mm, as confirmed from a scout image acquired with the surface coil. The constraint on lipid layer thickness ensured that the distance between the surface coil and the muscle tissue was consistent for all subjects. The calibration measurements were also acquired with approximately the same distance (4 mm, see below) between the measurement volume and the surface coil. These steps ensured that all signals were acquired from the same sensitive volume of the surface coil, which varies as a function of distance from the plane defined by the surface coil [37], [38]. Average in vivo metabolite concentrations were quantified for Pi, PCr, and ATP. The ATP concentration was determined from the average of the αATP and γATP moieties.

### Calibration measurements

Calibration sessions were required to determine the reference amplitudes, A_m,ref_ and A_p,ref_, in equation 1. The calibration data were acquired from a phantom containing a volumetrically-determined 75 mM sodium phosphate (NaH_2_PO_4_) concentration using the same pulse sequence and acquisition parameters used for the in vivo data. Agarose spacers with 4 mm thickness, approximately the same thickness as the subcutaneous lipid layer in all subjects, were placed between the phantom and the surface coil to ensure that the calibration measurements were acquired from the same sensitive volume of the surface coil as the in vivo measurements.

### Phantom replacement method

The phantom replacement method [30]–[33] was implemented using equation 1 after eliminating the two terms corresponding to the pseudo-signal, A_p_ and A_p,ref_. This quantification method assumes that RF coil loading conditions are the same for the calibration phantom measurements as they are for the in vivo measurements. This constraint was met by adjusting the salt (NaCl) concentration in the agarose spacer until the Q value of the surface coil was approximately the same as for the in vivo measurements. The empirically determined salt concentration that satisfied this constraint was 75 mM and the calibration data obtained during this session are referred to as the “properly loaded” calibration data.

To demonstrate immunity of the pFID measurements to differences in coil loading conditions, calibration data were also acquired using two additional agarose spacers. These spacers were also 4 mm thick but one contained no salt, and loaded the surface coil less than the in vivo measurements, and one contained 200 mM salt and loaded the surface coil more than the in vivo measurements. The calibration data obtained with no salt in the spacer are referred to as “underloaded”. The calibration data obtained with 200 mM salt in the spacer are referred to as “overloaded”. Hence, each metabolite concentration was calculated in six different ways; three calculations using the pFID method with calibration data obtained with an underloaded, properly loaded, and overloaded surface coil, and three corresponding concentrations using the phantom replacement method. The metabolite concentrations obtained using the phantom replacement method and the properly loaded calibration data are referred to as the nominal concentrations and were considered the gold standard against which all other results were compared.

## Results

**Figure 2:**
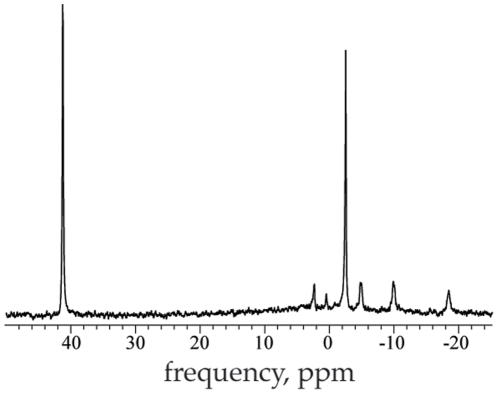
A sample in vivo spectrum with real peaks and the artificially injected pseudo-peak. The Pi, PCr and the γ, α, and β moieties of ATP appear at about 2.5, 0.5, −2.5, −5.0, −10, and −18.5 ppm, respectively. The pseudo-peak appears at 41 ppm. The amplitude, frequency, phase, and line-width of the pseudo-peak are determined by the digitized waveform transmitted by the RF synthesizer and are easily adjusted.

Figure 2 shows an example of an in vivo spectrum acquired from the tibialis anterior compartment of one subject. The real peaks corresponding to Pi, PCr and the γ, α, and β ATP moieties appear with sufficient signal to noise ratio (SNR) to allow reasonable estimates of their tissue content.

**Figure 3:**
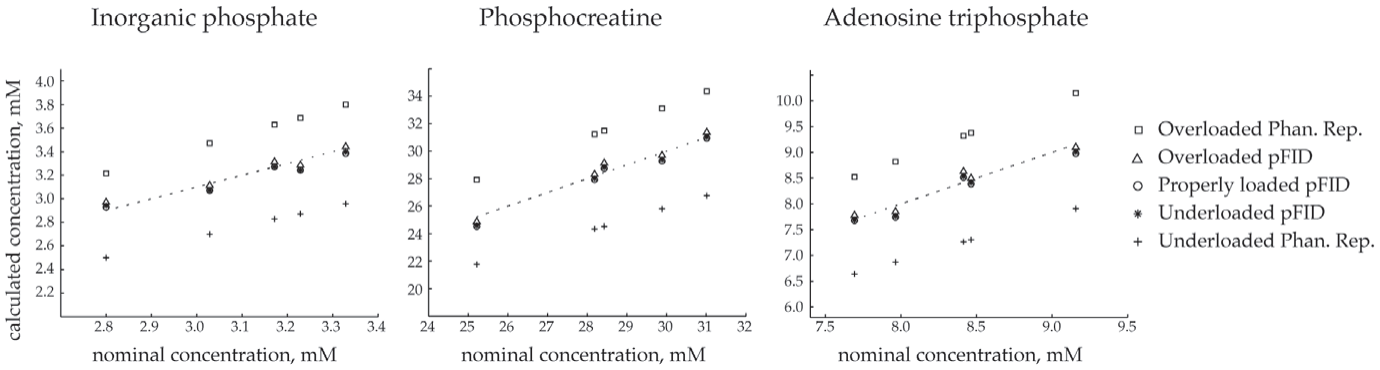
Concentrations determined by the pFID method are accurate and independent of coil loading conditions. The nominal concentrations (x-axes) were determined using the phantom replacement method and calibration measurements obtained from a phantom that replicated in vivo coil loading conditions. The nominal concentrations reflect the normal ranges of Pi, PCr and ATP expected in healthy male subjects. All three sets of pFID measurements fall very close to the lines of identity (dashed lines), validating that this method allows accurate quantification of metabolite content for a wide range of coil loading conditions. In contrast, the concentrations determined using the phantom replacement method were invalid when in vivo coil loading conditions were not replicated in the calibration session. When the calibration phantom overloaded the coil, the phantom replacement method overestimated the in vivo concentrations. When the phantom underloaded the coil, the phantom replacement method underestimated the in vivo concentrations.

Figure 3 shows the average Pi, PCr, and ATP concentrations within the measurement volume from all 5 subjects. The x-axes in the graphs are the nominal concentrations as determined using the phantom replacement method and the calibration measurements that were acquired with the surface coil loaded the same as it was for the in vivo measurements. The dashed lines represent the lines of identity. The concentrations determined using the pFID method all fall close to the line of identity, demonstrating that the calculations are independent of the coil loading conditions. The pFID values also fall well within the range of values reported in the literature [34]. In contrast, the concentrations determined using the phantom replacement method are highly dependent on coil loading conditions. When the surface coil was overloaded during the calibration session, the phantom replacement method consistently overestimated the metabolite concentrations by about 10%. When the surface coil was underloaded during the calibration session, the phantom replacement method consistently underestimated the concentrations by about 10%.

**Figure 4:**
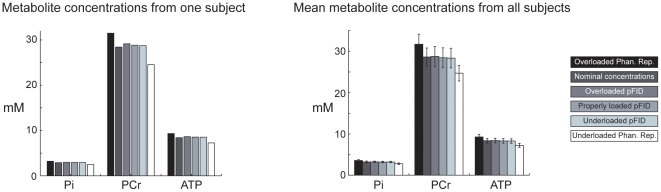
Typical metabolite concentrations from one subject and the mean (+/− standard deviation) metabolite concentrations from all subjects. The pFID method provided accurate estimates of metabolite content in each subject that were in agreement with the nominal concentrations. The pattern was consistent for all subjects, as reflected by the bar graph of the mean concentrations. The standard deviations primarily reflect the true range of concentrations within the subjects (as seen in Figure 3) rather than random variations in the measurements.

The same pattern is clear in the bar charts of Figure 4. When the pFID method was used, the calculated concentrations for individual subjects and the mean concentrations for all subjects were independent of coil loading conditions and agreed with the nominal concentrations. When the phantom replacement method was used, the calculated concentrations were strongly dependent on coil loading conditions.

## Discussion

Aside from techniques that employ the principle of reciprocity [39]–[41], the general algorithm most often used to convert MR spectra to units of concentration is to establish the calibration factor that describes the amplitude of the spectral peak generated by excited reference molecules at a known concentration, and then use that calibration factor to convert peaks of interest into units of concentration [42]–[44]. The reference signal can come from an internal substance in the tissue, such as water [45]–[49] or a metabolite [50], [51], whose concentration can be independently determined and remains stable during the course of the measurement. Internal reference signals can be acquired with the sample of interest properly positioned in the RF coil, which automatically compensates for coil loading. Unfortunately, there is no reliable reference signal that allows this method to be broadly applied to in vivo ^31^P measurements [41].

The reference signal can also come from an external substance present at a known concentration in a phantom. The external signal can be acquired from a vial placed outside the subject but within the sensitive volume of the RF coil [52]–[57]. This compensates for coil loading but is prone to problems with B_1_ sensitivity since the reference signal is necessarily acquired from a different location in the RF coil than the sample of interest. Also, the reference substance must resonate at a frequency that is distinct from the peaks of interest and this can be problematic in crowded spectra.

Problems with B_1_ sensitivity and overlap between peaks can be addressed by acquiring the external reference signal in a separate calibration session [30], [58]–[60]. This method is often referred to as the phantom replacement method or the replace and match method. A critical constraint for proper implementation is that the calibration phantom and the sample of interest must generate the same load on the RF coil. This is a difficult constraint to meet for human measurements because it requires accurate estimation of the in vivo loading conditions, which can vary widely, and the tedious fabrication of multiple phantoms that replicate them.

The results in Figures 3 and 4 demonstrate that the pFID method provides a robust calibration factor that is immune to coil loading conditions and allows accurate noninvasive assessment of metabolite concentrations in humans. The Pi, PCr, and ATP concentrations determined with the pFID method were consistent, in concordance with the nominal concentrations, and fell well within the range of normal values reported in the literature. In contrast, the concentrations determined with the phantom replacement method were clearly erroneous when in vivo coil loading conditions were not properly replicated during the calibration session. Immunity to coil loading conditions is perhaps the most important advantage of the pFID approach because it removes a substantial burden and potential source of error that is inherent with other MR quantification methods.

For this developmental study, our subject pool was limited to those with a specific lipid layer thickness and therefore the same offset distance between the RF coil and the muscle tissue. This constraint was imposed because we used a surface coil, and therefore an inherently nonuniform RF field, to acquire the data. Our limited subject pool ensured that all the in vivo metabolite signals were acquired from the same sensitive volume of the RF coil as the calibration data acquired from the reference phantom. This constraint could be removed by using an acquisition coil with a sufficiently large homogenous region to allow volume localization or by conducting additional calibration measurements with a range of offsets from the acquisition coil. To allow straightforward validation, our data were acquired using a simple pulse-acquire sequence with a long repetition time, precluding the need to correct for relaxation/saturation effects. More efficient data acquisition and more complicated pulse sequences could be accommodated if additional relaxation measurements were acquired to allow correction for T1 and T2 effects.

Our results demonstrate that the pFID method allows expedient and accurate determination of in vivo metabolite content. The protocol we describe could be readily adapted for use in pediatric patients with suspected mitochondrial disorder, where marked alterations in phosphorous metabolites have been observed [61]–[63]. Robust ^31^P-MRS measurements in muscle could be used to monitor therapy in these patients [64], after establishing the normal pattern of variation during development [65], [66]. The pFID method is also applicable for other MR-visible nuclei. We are currently building a pFID probe for use in human brain measurements, where quantitative MRS could enhance the ability to distinguish tumor types [67], [68] and assess neurotoxicity following radiation treatment [69]. We also plan to build a ^19^F probe, which could be used to determine whole brain fluorine content in subjects being treated with fluorinated antidepressant or antipsychotic medications.

The main disadvantage to using inductive coupling for injecting the pseudo-signal is that it currently requires a specially modified RF probe that includes an injector coil. While the cost of the modification is relatively low, this might restrict its use to research sites that have the required expertise. To address this, we are currently exploring the feasibility of using a detachable injector coil that would be compatible with multiple RF coils and would reduce the burden of implementation.
